# *Acinetobacter baumannii* Outer Membrane Vesicles Elicit a Potent Innate Immune Response via Membrane Proteins

**DOI:** 10.1371/journal.pone.0071751

**Published:** 2013-08-14

**Authors:** So Hyun Jun, Jung Hwa Lee, Bo Ra Kim, Seung Il Kim, Tae In Park, Je Chul Lee, Yoo Chul Lee

**Affiliations:** 1 Department of Microbiology, Kyungpook National University School of Medicine, Daegu, Korea; 2 Division of Life Science, Korea Basic Science Institute, Daejeon, Korea; 3 Department of Pathology, Kyungpook National University School of Medicine, Daegu, Korea; University of Florida, College of Dentistry & The Emerging Pathogens Institute, United States of America

## Abstract

*Acinetobacter baumannii* is increasingly becoming a major nosocomial pathogen. This opportunistic pathogen secretes outer membrane vesicles (OMVs) that interact with host cells. The aim of this study was to investigate the ability of *A. baumannii* OMVs to elicit a pro-inflammatory response *in vitro* and the immunopathology in response to *A. baumannii* OMVs *in vivo*. OMVs derived from *A. baumannii* ATCC 19606^T^ induced expression of pro-inflammatory cytokine genes, interleukin (IL)-1β and IL-6, and chemokine genes, IL-8, macrophage inflammatory protein-1α, and monocyte chemoattractant protein-1, in epithelial cells in a dose-dependent manner. Disintegration of OMV membrane with ethylenediaminetetraacetic acid resulted in low expression of pro-inflammatory cytokine genes, as compared with the response to intact OMVs. In addition, proteinase K-treated *A. baumannii* OMVs did not induce significant increase in expression of pro-inflammatory cytokine genes above the basal level, suggesting that the surface-exposed membrane proteins in intact OMVs are responsible for pro-inflammatory response. Early inflammatory processes, such as vacuolization and detachment of epithelial cells and neutrophilic infiltration, were clearly observed in lungs of mice injected with *A. baumannii* OMVs. Our data demonstrate that OMVs produced by *A. baumannii* elicit a potent innate immune response, which may contribute to immunopathology of the infected host.

## Introduction

*Acinetobacter baumannii* is a Gram-negative, lactose non-fermenting aerobic coccobacillus and an important opportunistic pathogen that causes various types of infections, including ventilator-associated pneumonia, urinary tract infection, skin and wound infections, bacteremia, and meningitis [1]. This microorganism is regarded as a low virulence pathogen, but increasing evidence has highlighted the importance of *A. baumannii* as a nosocomial pathogen responsible for high morbidity and mortality of infected patients, especially in severely ill patients [1], [2]. Clinical significance of *A. baumannii* has also increased due to its ability to develop antimicrobial resistance to currently available antimicrobial agents, which causes a serious therapeutic problem [2]–[4].

A number of virulence traits of *A. baumannii*, such as biofilm formation [5], [6], adherence and invasion to host cells [7], [8], serum resistance [9], and host cell death [10], [11], have been characterized, however, much less is known regarding the immune responses to *A. baumannii* that are critical to disease development. An innate immune response against *A. baumannii* via sensing of lipopolysaccharide (LPS) through CD14 and Toll-like receptor (TLR) 4 effectively eliminated bacteria from the lungs in a mouse pneumonia model, whereas TLR2 signaling counteracted the robustness of innate immune responses [12], [13]. Breij et al [14] recently reported an association of the outcome of *A. baumannii*-induced pneumonia with anti-inflammatory interleukin (IL)-10 and pro-inflammatory IL-12p40 and IL-23 cytokine levels in a mouse pneumonia model. However, little is known with regard to the interaction of *A. baumannii*-derived secretory products with host cells leading to the innate immune response.

Gram-negative pathogens secrete outer membrane vesicles (OMVs), which are recognized as delivery vehicles for bacterial effectors to host cells [15]–[19]. OMVs are spherical nanovesicles with an average diameter of 20 - 200 nm and are composed of LPS, proteins, lipids, and DNA or RNA [16], [17]. OMVs produced by Gram-negative pathogens transport diverse virulence factors to host cells simultaneously and allow interaction of pathogens with the host without close contact between bacteria and host cells [20]. In addition, OMVs contain adhesins, invasins, toxins, and pathogen-associated molecular patterns (PAMPs) and they can contribute to bacterial pathogenesis and immunopathology in the host. We previously demonstrated that *A. baumannii* OMVs contain multiple virulence factors, including outer membrane protein A (AbOmpA), proteases, phospholipases, superoxide dismutase, and catalase [21]. Of particular interest, *A. baumannii* OMVs interact with host cells and then deliver bacterial effectors to host cells via lipid rafts, resulting in cytotoxicity [22]. However, immune response to *A. baumannii* OMVs has not yet been characterized. The aim of this study was to investigate an innate immune response to *A. baumannii* OMVs in both *in vitro* cultured epithelial HEp-2 cells and an *in vivo* mouse model. We report here that *A. baumannii* OMVs are potent stimulators of inflammatory response both *in vitro* and *in vivo*.

## Materials and Methods

### Bacterial Strain

*A. baumannii* ATCC 19606^T^ was used for preparation of OMVs and infected cells. AbOmpA-deficient mutant KS37 strain was also used for preparation of OMVs [10]. *A. baumannii* ATCC 19606^T^ was provided by Lenie Dijkshoorn (Leiden University Medical Center, The Netherlands) and bacteria were grown in Luria-Bertani (LB) broth.

### Cell Culture

Human laryngeal epithelial HEp-2 cells were obtained from Korean Cell Line Bank (Seoul, Korea) and were grown in Dulbecco’s modified Eagle medium (HyClone) supplemented with 10% fetal bovine serum (HyClone), 2 mM _L_-glutamine, 1,000 U/ml penicillin G, and 50 µg/ml streptomycin at 37°C in 5% CO_2_. Confluent cells were harvested and seeded into wells of 96-well plates for the cell viability assay and 6- (4×10^5^ cells/well) or 12-well (5×10^4^ cells/well) plates for the cytokine gene assay. HEp-2 cells were treated with OMVs, live or formalin-fixed bacteria, or phosphate-buffered saline (PBS), and incubated until time of assay.

### Purification of OMVs

OMVs produced by *A. baumannii* were prepared as previously described [22], [23]. Briefly, *A. baumannii* ATCC 19606^T^ was grown in 500 ml of LB broth to reach late log phase at 37°C with shaking. Bacterial cells were removed by centrifugation at 6,000 × g for 20 min at 4°C. The supernatants were filtered using a QuixStand Benchtop System (GE Healthcare) using a 0.2 µm-sized hollow fiber membrane (GE Healthcare) and then concentrated using a QuixStand Benchtop System using a 100 kDa hollow fiber membrane (GE Healthcare). After ultrafiltration of OMVs, the samples were collected by ultracentrifugation at 150,000 × g for 3 h at 4°C and resuspended in PBS. The protein concentration was determined using the modified BCA assay (Thermo Scientific). The purified OMVs were checked to sterility and stored at −80°C until used.

### Treatment of OMVs with Proteinase K and Ethylenediaminetetraacetic Acid (EDTA)

Purified OMVs were treated with 0.1 µg/ml of proteinase K (Fermentas) for 1 h at 37°C for degradation of surface-exposed proteins in the OMVs and 0.1 M EDTA for 1 h at 37°C for disintegration of OMV membrane. OMV samples, including intact OMVs and proteinase K- and EDTA-treated OMVs, were separated by 12% sodium dodecyl sulfate-polyacrylamide gel electrophoresis and stained with Coomassie brilliant blue R-250 (Bio-Rad).

### RNA Extraction and Quantitative Real-time Polymerase Chain Reaction (PCR)

HEp-2 cells were treated with *A. baumannii* OMVs (1–15 µg/ml of media) or infected with live or formalin-fixed *A. baumannii* with multiplicity of infection (MOI) 1–300 for 24 h. Total cellular RNA was harvested using the Qiagen RNeasy kit according to the manufacturer’s instructions. The RNA samples were treated with DNase (Qiagen) for removal of contaminating DNA. Harvested RNA was quantitated using a spectrophotometer (Bio-Rad). cDNA was generated by reverse transcription of 1 µg of total RNA using oligo dT primers and M-MLV reverse transcriptase in a total reaction volume of 20 µl (Fermentas). The reaction mixtures were incubated for 1 h at 37°C and the samples were stored at −20°C. For quantitative real-time PCR, primer sequences were designed using Primer Express Software (version 3.0) (Applied Biosystems). The primer sequences of target genes were 5′-GGA CCT GAC CTG CCG TCT AG-3′ and 5′- GAG GAG TGG GTG TCG CTG TT-3′ for glyceraldehydes 3-phosphate dehydrogenase (GAPDH), 5′-CCT GTC CTG CGT GTT GAA AGA-3′ and 5′-GGG AAC TGG GCA GAC TCA AA-3′ for IL-1β, 5′-TGG CTG AAA AAG ATG GAT GCT-3′ and 5′-TCT GCA CAG CTC TGG CTT GT-3′ for IL-6, 5′-TTG GCA GCC TTC CTG ATT TC-3′ and 5′-TGG TCC ACT CTC AAT CAC TCT CA-3′ for IL-8, 5′-ATC GTC CAC GCC GTG TTT-3′ and 5′-GCT GCA GGT GTG GTG AGT GA-3′ for macrophage inflammatory protein (MIP)-1α, and 5′-TCG CTC AGC CAG ATG CAA T-3′ and 5′-TGG CCA CAA TGG TCT TGA AG-3′ for monocyte chemoattractant protein (MCP)-1. Real-time PCR was performed using an ABI PRISM 7500 Real-Time System using the Power SYBR Green PCR Master Mix (Applied Biosystems). The amplification specificity was evaluated using melting curve analysis. Gene expression was normalized to GAPDH mRNA levels in each sample and fold change was determined using the ΔΔCt method [24].

### TEM Analysis

The purified OMV samples were applied to copper grids and stained with 2% uranyl acetate. The samples were visualized on a transmission electron microscope (Hitachi H-7500, Hitachi, Japan) operating at 120 kV.

### Cell Viability

The cytotoxicity of HEp-2 cells treated with *A. baumannii* OMVs was measured using the Premix WST1 cell proliferation assay system (Takara, Japan) [10]. The cells were seeded at a concentration of 2.0 × 10^5^/ml in 96-well microplates. After treatment with different concentrations of OMVs, cellular cytotoxicity was measured at 450 nm 3 h after treatment with WST1.

### Mouse Inflammation Model

Female Balb/c mice (eight weeks old) were maintained under specific pathogen free conditions. For induction of inflammatory response to *A. baumannii* OMVs in the skin, mice received intradermal injection of OMVs (200 µg of OMVs suspended in 100 µl of PBS). For assessment of inflammatory response to *A. baumannii* OMVs in the lungs, mice were anesthetized with Avertin (Sigma) and OMVs (200 µg of OMVs suspended in 100 µl of PBS) were administered intratracheally [11]. The control mice were injected with 100 µl of PBS (pH 7.4) in both experiments. Mice were sacrificed 24 h after OMV challenge. Skin and lung tissues were stained with hematoxilin and eosin (H & E). The animal experimental procedures were approved by the Animal Care Committee of Kyungpook National University (KNU2012-5).

## Results

### *A. baumannii* OMVs Elicit a Pro-inflammatory Response in Epithelial Cells

**Figure 1:**
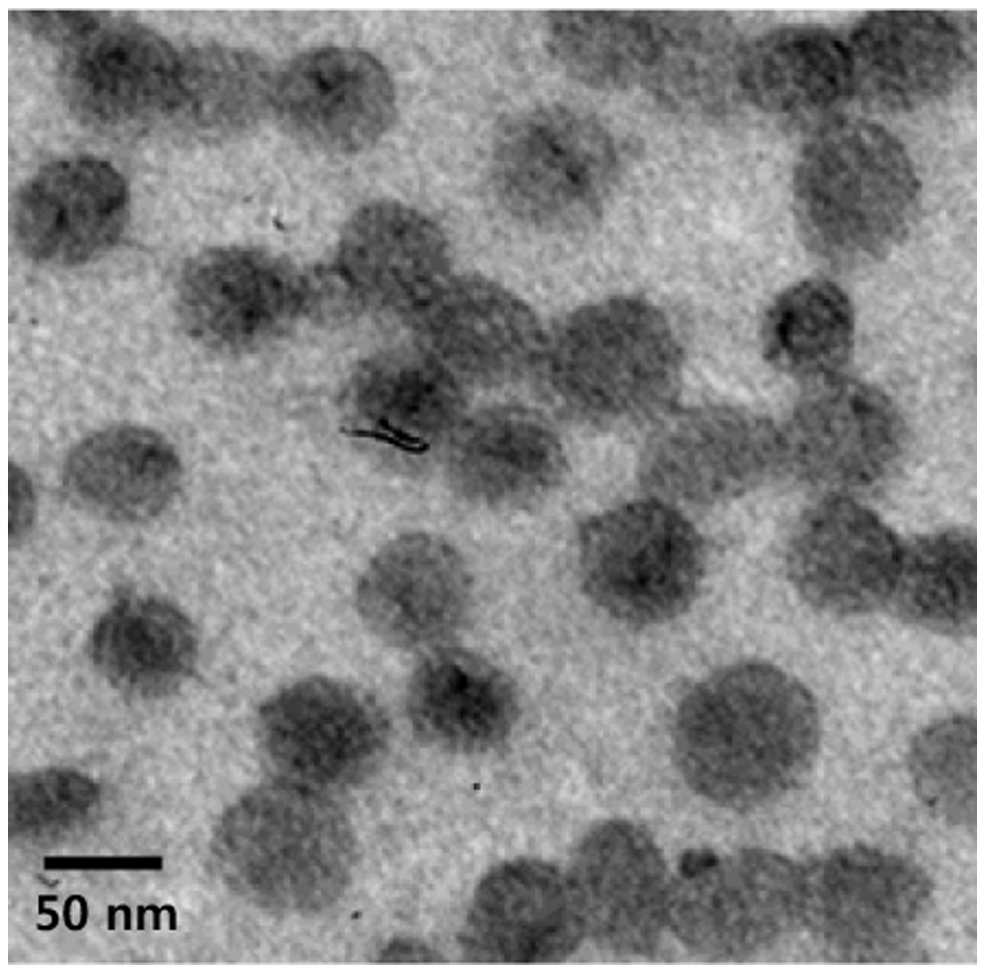
Transmission electron micrograph of OMVs prepared from *A. baumannii* ATCC 19606^T^ cultured in LB broth.

**Figure 2:**
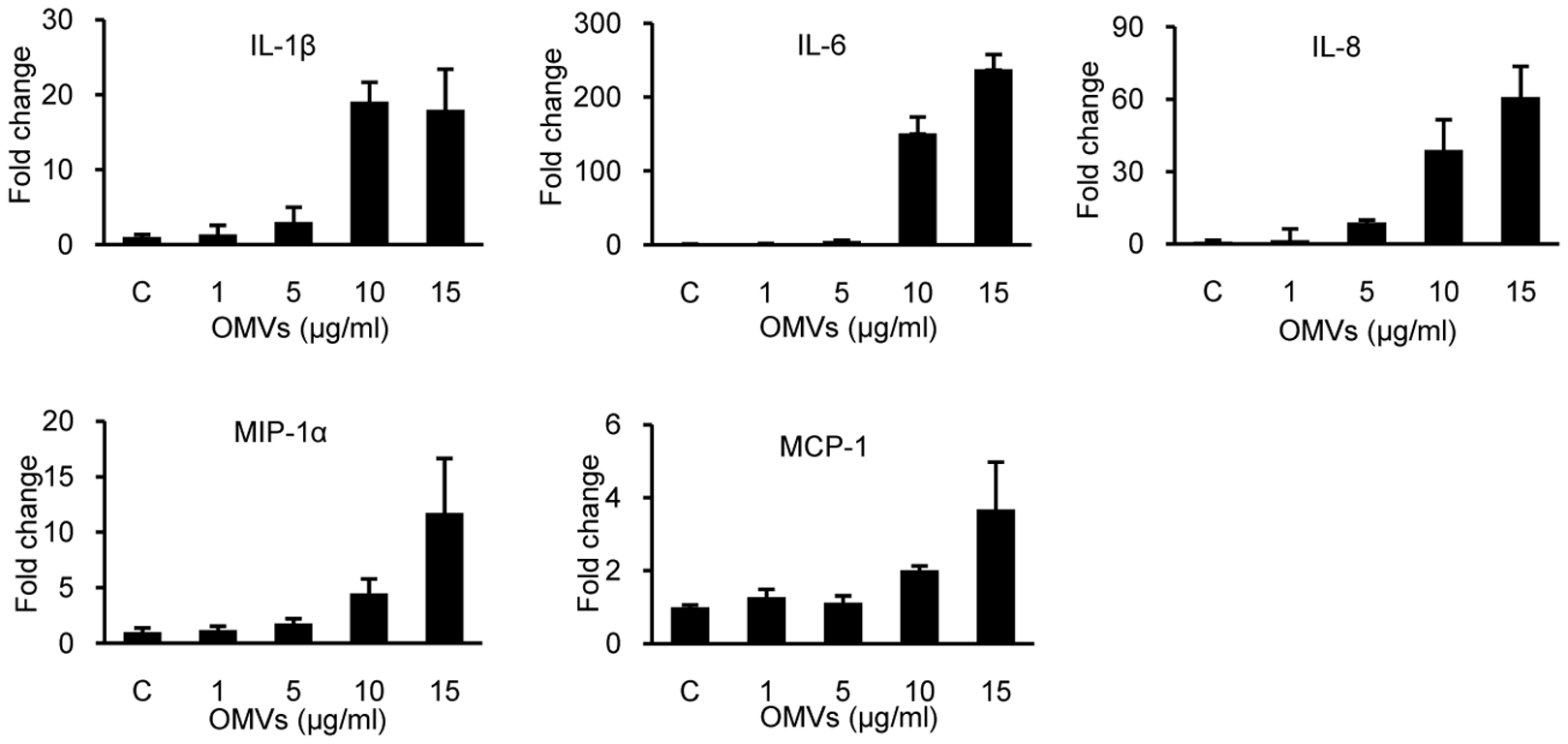
The pro-inflammatory response of HEp-2 cells to *A. baumannii* OMVs. HEp-2 cells were treated with different concentrations (1–15 µg/ml) of *A. baumannii* OMVs for 24 h. Gene expression was assessed by quantitative real-time PCR, as described in the Materials and Methods section. Data are presented as mean ± SD of duplicate determinations.

OMVs were purified from the culture supernatant of *A. baumannii* ATCC 19606^T^ and TEM analysis was performed. *A. baumannii* OMVs were spherical bilayered nanovesicles and maintained membrane integrity (Fig. 1). Because OMVs from *A. baumannii* ATCC 19606^T^ induced cytotoxicity in macrophage U937 cells [22], the cytotoxic activity of the purified OMVs was determined in HEp-2 cells. Cultured HEp-2 cells were treated with various concentrations (1–50 µg/ml) of *A. baumannii* OMVs for 24 h and cellular damage was assessed using inverted microscopy and WST1 cell proliferation assay. Cytotoxicity of HEp-2 cells was not observed in response to ≤15 µg/ml of *A. baumannii* OMVs, however, ≥20 µg/ml of OMVs induced cytotoxicity such as cellular shrinkage, rounding of cells, and cell detachment from the bottom. Results of the WST1 assay showed that ≥20 µg/ml of *A. baumannii* OMVs also induced cytotoxicity of HEp-2 cells (data not shown). Next, in order to determine whether *A. baumannii* OMVs could trigger a pro-inflammatory response, HEp-2 cells were treated with sublethal doses of *A. baumannii* OMVs (1–15 µg/ml) and quantitative real-time PCR was performed for analysis of expression of pro-inflammatory cytokine genes, including IL-1β, IL-6, IL-8, MIP-1α, and MCP-1. *A. baumannii* OMVs stimulated significant transcription of all pro-inflammatory cytokine genes tested (Fig 2). Expression of pro-inflammatory cytokine genes, except IL-1β, was clearly dose-dependent and showed a sharp increase in response to treatment with 10 µg/ml of *A. baumannii* OMVs.

### Comparison of Pro-inflammatory Cytokine Response in HEp-2 cells Treated with *A. baumannii* and its Derived OMVs

**Figure 3:**
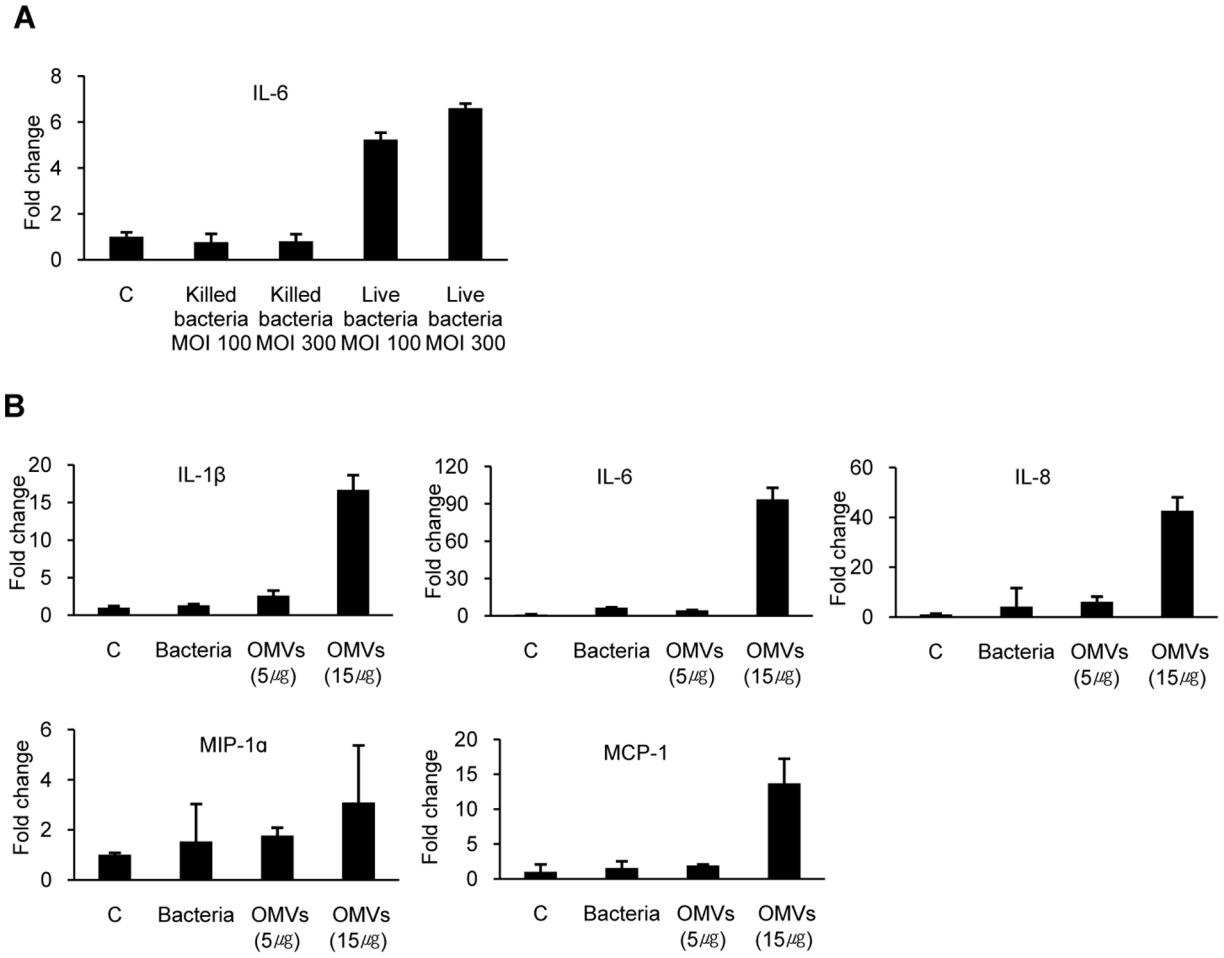
The pro-inflammatory response of HEp-2 cells to *A. baumannii* and OMVs. (A) HEp-2 cells were infected with live or formalin-fixed *A. baumannii* with MOI 100 and 300 for 24 h. (B) HEp-2 cells were infected with *A. baumannii* with MOI 300 and treated with 5 or 15 µg/ml of *A. baumannii* OMVs for 24 h. Gene expression of pro-inflammatory cytokines was assessed by quantitative real-time PCR. Data are presented as mean ± SD of duplicate determinations.

In order to determine the pro-inflammatory response to *A. baumannii*, HEp-2 cells were treated with live or formalin-fixed *A. baumannii* with MOI 1–300 for 24 h and expression of IL-6 gene was measured. Expression of IL-6 gene was not increased in either live *A. baumannii* with MOI 10 (data not shown) or formalin-fixed bacteria with MOI up to 300 (Fig 3A). Treatment with live *A. baumannii* with MOI 100 and 300 resulted in stimulation of IL-6 gene expression in HEp-2 cells. To compare expression of pro-inflammatory cytokine genes in host cells respond to *A. baumannii* infection and OMV treatment, HEp-2 cells were infected with *A. baumannii* with MOI 300 or treated with 5 or 15 µg/ml of OMVs. Expression level of pro-inflammatory cytokine genes in HEp-2 cells infected with live bacteria was similar to that of cells treated with 5 µg/ml of OMVs (Fig 3B). However, 15 µg/ml of *A. baumannii* OMVs elicited profoundly greater pro-inflammatory cytokine response than that observed in response to live *A. baumannii* with MOI 300.

### Surface-exposed Membrane Proteins in *A. baumannii* OMVs are Responsible for Pro-Inflammatory Cytokine Response

**Figure 4:**
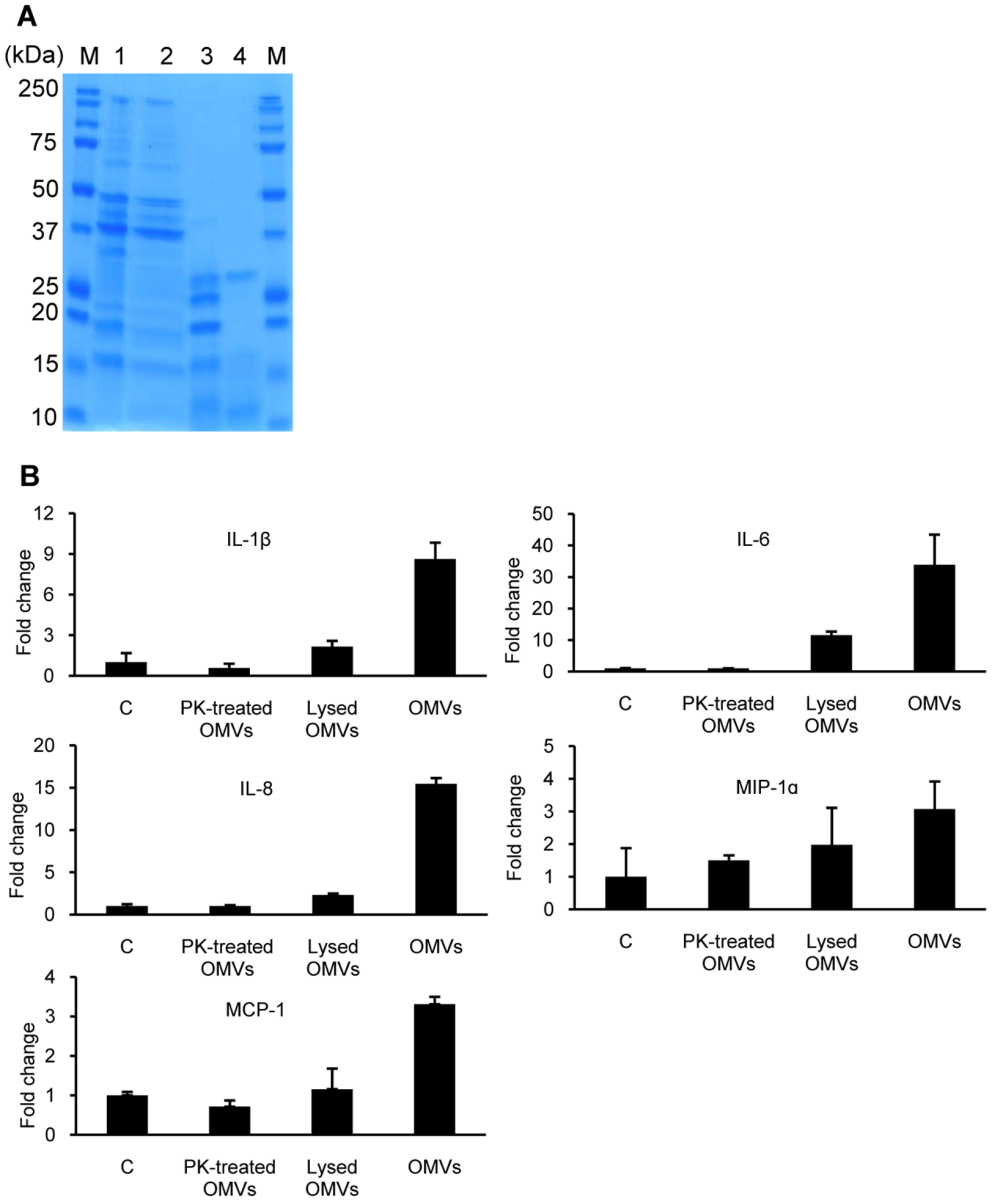
The pro-inflammatory response of HEp-2 cells to *A. baumannii* OMVs treated with proteinase K and EDTA. (A) Protein profiles of *A. baumannii* OMVs. Lane M, size marker; 1, purified intact OMVs; 2, OMVs treated with EDTA; 3, OMVs treated with proteinase K; 4, proteinase K. (B) Expression of pro-inflammatory cytokine genes to proteinase K- and EDTA-treated *A. baumannii* OMVs was assessed by quantitative real-time PCR. HEp-2 cells were treated with the same concentration (15 µg/ml) of *A. baumannii* OMVs for 24 h as a positive control. Data are presented as mean ± SD of duplicate determinations.

**Figure 5:**
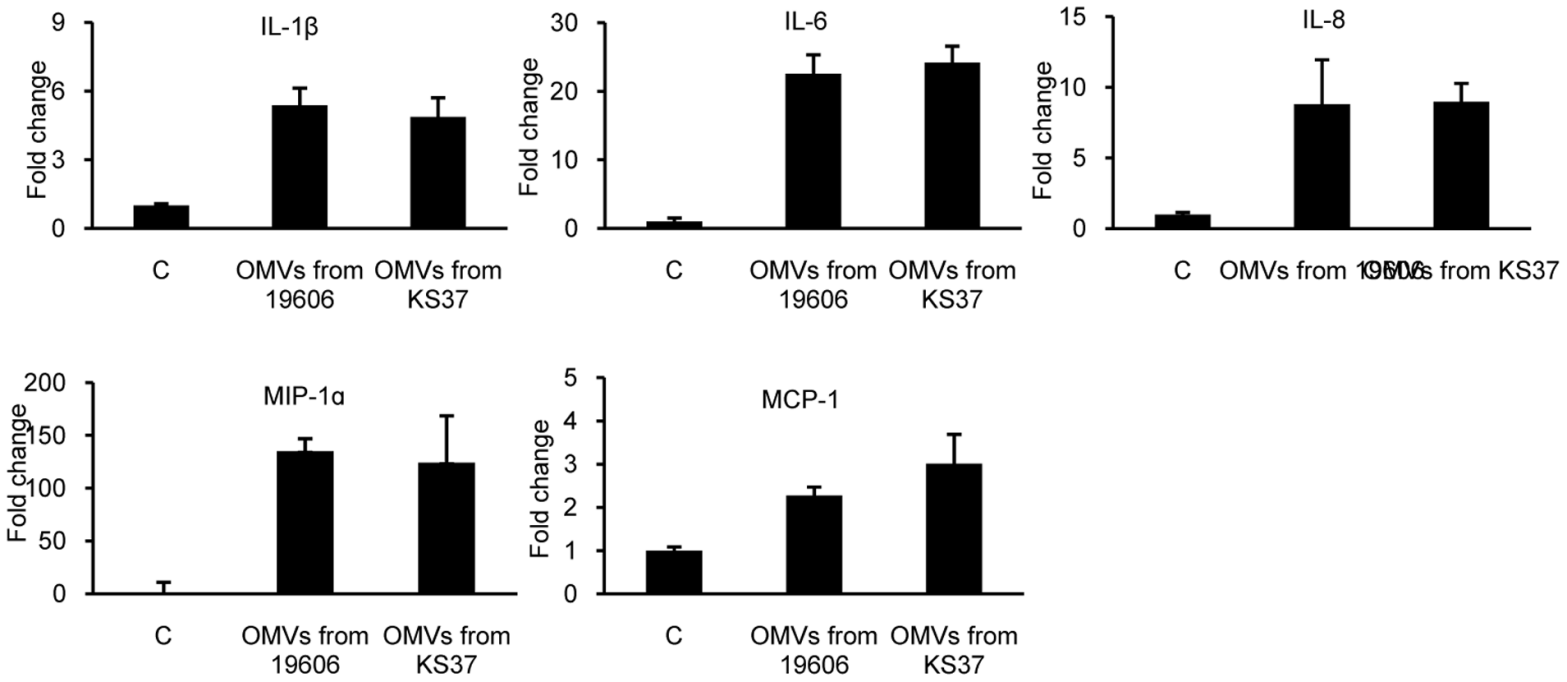
The pro-inflammatory response of HEp-2 cells to OMVs from *A. baumannii* ATCC 19606^T^ and its isogenic AbOmpA-deficient mutant KS37 strains. HEp-2 cells were treated with the same concentration (15 µg/ml) of OMVs for 24 h. Gene expression of pro-inflammatory cytokines was assessed by quantitative real-time PCR. Data are presented as mean ± SD of duplicate determinations.

To determine which OMV components are responsible for pro-inflammatory cytokine response, HEp-2 cells were treated with proteinase K-treated *A. baumannii* OMVs and gene expression of pro-inflammatory cytokines was measured. Treatment with proteinase K resulted in alteration of the protein profile of OMVs (Fig 4A). All proteins in the OMVs were not degraded, but many high molecular weight bands disappeared, suggesting degradation of membrane proteins and protection of luminal proteins. Up-regulation of pro-inflammatory cytokine genes was not observed in response to proteinase K-treated *A. baumannii* OMVs (Fig. 4B). Next, in order to determine whether lysed OMVs stimulated pro-inflammatory response like that of intact OMVs, *A. baumannii* OMVs were pre-treated with EDTA in order to disintegrate the OMV membrane, resulting in lysis of OMVs, and HEp-2 cells were treated with lysed OMVs for 24 h. Treatment with EDTA did not result in alteration of the protein profile of OMVs (Fig 4A). The lysed *A. baumannii* OMVs induced up-regulation of IL-1β, IL-6, IL-8, and MIP-1α genes, but not induce MCP-1 gene (Fig. 4B). Intact *A. baumannii* OMVs elicited greater pro-inflammatory cytokine gene expression than the response to the lysed *A. baumannii* OMVs (Fig 4B). To determine whether AbOmpA in *A. baumannii* OMVs was responsible for pro-inflammatory cytokine response, HEp-2 cells were treated with OMVs purified from *A. baumannii* ATCC 19606^T^ and its isogenic AbOmpA-deficient mutant KS37 strain for 24 h. Both OMVs from wild-type and AbOmpA mutant strains up-regulated gene expression of pro-inflammatory cytokines; however, there was no significant difference in expression of pro-inflammatory cytokine genes between OMVs from wild-type and AbOmpA mutant strains (Fig 5).

### *A. baumannii* OMVs Induce Inflammatory Response *in vivo*

**Figure 6:**
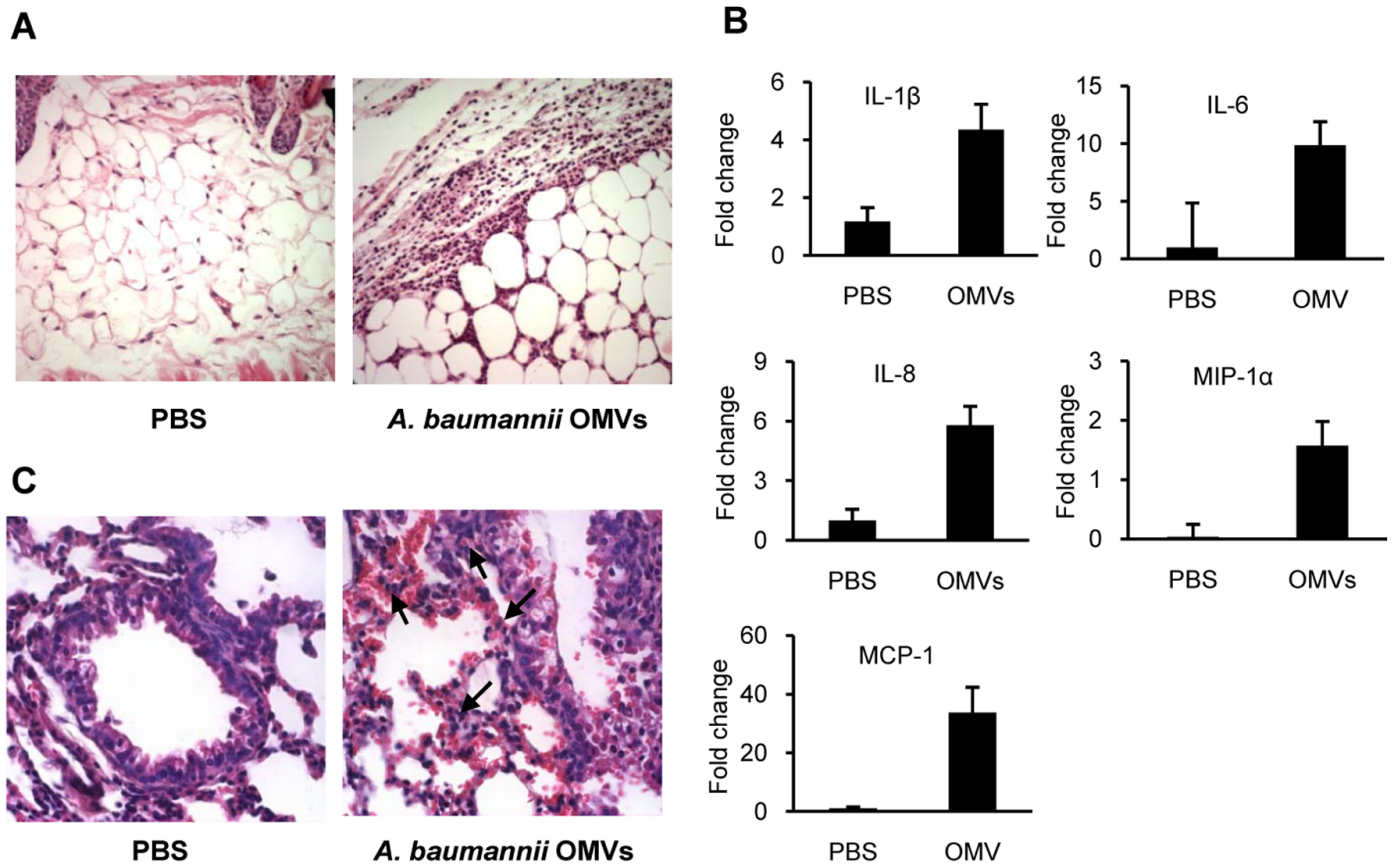
The inflammatory response to *A. baumannii* OMVs *in vivo*. (A) Recruitment of neutrophils in mouse skin. Mice received intradermal administration of *A. baumannii* OMVs in the back and skin lesions were stained with hematoxylin and eosin. PBS was administered as a control. Magnification, ×100. (B) The pro-inflammatory cytokine response of lung tissues to *A. baumannii* OMVs. Mice were treated with *A. baumannii* OMVs or PBS for 24 h and the lung tissues were removed. Gene expression was assessed by quantitative real-time PCR. Data are presented as mean ± SD of three mice. (C) Early inflammatory response in the lungs. *A. baumannii* OMVs were administered intratracheally and the lung tissues were stained with H & E. PBS was administered as a control. Arrows indicate neutrophils. Magnification, ×400.

In order to determine whether *A. baumannii* OMVs could induce an inflammatory response *in vivo*, mice received intradermal injection of *A. baumannii* OMVs in the back and the inflammatory response was determined. As shown in Fig 6A, massive neutrophilic infiltration was observed in the skin. Because the respiratory tract was the most common site of *A. baumannii* infection, we determined the ability of *A. baumannii* OMVs to elicit an inflammatory response in the lungs. *A. baumannii* OMVs were administered intratracheally and lungs were removed from mice. *A. baumannii* OMVs also elicited pro-inflammatory cytokine genes, including IL-1β, IL-6, IL-8, MIP-1α, and MCP-1, in the lungs (Fig. 6B). In addition, early inflammatory processes, including congestion, hemorrhage, vacuolization and detachment of bronchiolar epithelial cells, and neutrophilic infiltration, were clearly observed in lungs of mice injected with *A. baumannii* OMVs (Fig. 6C).

## Discussion

Several reports have described the production, proteomic analysis, functional roles in host cells, and vaccine trial of *A. baumannii* OMVs [21], [22], [25], [26], however, little is known about the innate immune response to *A. baumannii* OMVs associated with immunopathology. Thus, we investigated the innate immune response to *A. baumannii* OMVs both *in vitro* and *in vivo*. Our data demonstrate that *A. baumannii* OMVs elicit a pro-inflammatory response via surface-exposed membrane proteins *in vitro* and trigger a potent inflammatory response *in vivo*.

We have previously shown that *A. baumannii* ATCC 19606^T^ and ATCC 17978 produce OMVs during both *in vitro* culture and *in vivo* mouse infection [22]. Koning et al. [27] recently reported that *A. baumannii* 19606^T^ produces morphologically different types of OMVs during the various stages of bacterial culture. Regular-shaped and small-sized vesicles are produced by log phase bacteria, whereas deformed and large-sized vesicles are produced by stationary phase bacteria. OMVs derived from different stages of bacterial culture may show compositional difference. In this study, purified OMVs from *A. baumannii* ATCC 19606^T^ exhibited a regular shape and sized with 40–70 nm (Fig 1), suggesting that *A. baumannii* OMVs obtained in this study originated from log phase bacteria. Because OMVs from *A. baumannii* 19606^T^ were proven to contain cytotoxic proteins such as AbOmpA and the interaction of *A. baumannii* OMVs with host cells induced host cell damage [22], we determined the cytotoxicity of *A. baumannii* OMVs in HEp-2 cells. In the previous study, ≥50 µg/ml of *A. baumannii* OMVs induced cytotoxicity in macrophage U937 cells. However, in this study, ≥20 µg/ml of *A. baumannii* OMVs induced cytotoxicity of HEp-2 cells. Discrepancy in amounts of *A. baumannii* OMVs for induction of cell death may be due to the different bacterial culture stages for OMV purification, which may result in compositional difference of the purified OMVs.

An innate immune response is accompanied by bacterial colonization and infection. OMVs produced by Gram-negative bacteria contain various PAMPs, such as LPS, outer membrane porins, flagellins, peptidoglycans, and DNA [16], [19]. These immune activating ligands in OMVs interact with and are internalized by neighboring epithelial cells and immune cells [18], [19], [28]. The potency of OMVs in triggering an innate immune response was evident by several pathogens, such as *Pseudomonas aeruginosa* [20], [29] and *Salmonella enterica* serovar Typhimurium [30]. OMVs stimulate expression of major histocompatibility complex, production of pro-inflammatory cytokines and chemokines, and synthesis of nitric oxide in professional antigen presenting cells and epithelial cells, leading to inflammatory response in the hosts. OMVs from *A. baumannii* ATCC 19606^T^ have also been reported to carry a variety of PAMPs, such as porins, other outer membrane proteins, and LPS, like OMVs from other Gram-negative bacteria [22], [25]. In this study, the ability of *A. baumannii* OMVs to elicit a pro-inflammatory response was determined. Our data clearly demonstrate that *A. baumannii* OMVs are potent stimulators of pro-inflammatory cytokines, including IL-1β, IL-6, IL-8, MIP-1α, and MCP-1, in epithelial cells (Fig 2). We determined gene expression of pro-inflammatory cytokines in response to live *A. baumannii* infection. Expression level of pro-inflammatory cytokine genes in HEp-2 cells infected with *A. baumannii* with MOI 300 was comparable to that of cells treated with 5 µg/ml of OMVs (Fig 3B). Although OMV concentrations for treatment of cells are a relatively high to obtain it *in vitro* culture condition, OMV production is associated with bacteria stress condition and is increased under harsh conditions [25], [31]. These results suggest that OMVs produced by *A. baumannii* can induce pro-inflammatory response during *in vivo* infection.

OMVs derived from Gram-negative pathogens play a role as protective transport vehicles, delivering bacterial effector molecules such as toxins, enzymes, and DNA to host cells [19]. *A. baumannii* OMVs bind to the cytoplasmic membrane of host cells and deliver bacterial effectors to host cells [22]. In this study, intact OMVs elicited a pro-inflammatory response in a dose-dependent manner, whereas gene expression of pro-inflammatory cytokines in response to lysed OMVs did not reach that of intact OMVs (Fig 4B). This result suggests that OMV-mediated delivery of bacterial effectors is critical to induction of pro-inflammatory response. In addition, pro-inflammatory response to proteinase K-treated OMVs did not induce expression of cytokine genes, although the luminal proteins were conserved in the OMVs, confirming the role of surface-exposed membrane proteins in triggering a pro-inflammatory response. AbOmpA, an abundant protein in *A. baumannii* OMVs, is known as a specific virulence factor [10]. AbOmpA contributes directly or indirectly to multiple aspects of *A. baumannii* pathogenesis through biofilm formation [32], serum resistance [9], host cell cytotoxicity [10], [11], and adherence and invasion of host cells [8]. These results may suggest that AbOmpA plays a role in pro-inflammatory response. However, our results showed that AbOmpA packaged in *A. baumannii* OMVs did not exert on the expression of pro-inflammatory cytokine genes in HEp-2 cells (Fig 5). Furthermore, recombinant AbOmpA (rAbOmpA) did not induce any pro-inflammatory cytokine in HEp-2 cells, although rAbOmpA induced gene expression of TLR 2 and inducible nitric oxide synthase [33]. Future studies will focus on determining which membrane proteins are critical to the observed pro-inflammatory responses.

We have previously shown that *A. baumannii* induced an inflammatory response such as recruitment of inflammatory cells and exudates in the lungs of neutropenic mice. Neutrophil recruitment and activation are important for host defense to systemic *A. baumannii* infection [34]. Our data showed that *A. baumannii* OMVs recruited neutrophils in skin of mice. After confirming inflammatory properties of *A. baumannii* OMVs, we determined pulmonary inflammation in mice administered with *A. baumannii* OMVs. *A. baumannii* OMVs induced early inflammatory response in the lungs, but inflammatory response in the lungs was weak, as compared to skin lesions injected with *A. baumannii* OMVs. Our data highlight the potential inflammatory consequences of OMVs produced by *A. baumannii* during colonization or infection. Future studies should be conducted in order to determine whether the innate immune response to *A. baumannii* OMVs can stimulate clearance of bacteria or enhance pathogenic potential of bacteria.

In conclusion, the data presented here demonstrate that *A. baumannii* OMVs are potent stimulators of innate immune response and that membrane proteins in OMVs are critical for induction of an innate immune response. Epithelial response to *A. baumannii* OMVs may explain in part the innate immune response during colonization or early infection of *A. baumannii*.
